# High-throughput metagenomics analysis in early and precise diagnosis of eosinophilic meningoencephalitis complicated with respiratory failure: A case report

**DOI:** 10.1097/MD.0000000000033683

**Published:** 2023-05-12

**Authors:** Guoqing Qiu, Liqun Zhu, Sida Chen, Na Li, Chunxing Ye, Songming Zhuo, Yan Shen

**Affiliations:** a Respiratory Department, Longgang Central Hospital, Shenzhen, China; b Otolaryngological Department, South China Hospital of Shenzhen University, Shenzhen, China.

**Keywords:** *Angiostrongyliasis cantonensis*, case report, eosinophilic meningoencephalitis, High-throughput metagenomics

## Abstract

Human infection with *Angiostrongylus cantonensis* is uncommon and has only been sporadically reported in the literature. Patients infected with *A cantonensis* usually have a delayed diagnosis and sometimes a poor prognosis.

**Patient concerns:**

A 70-year-old woman presented to the respiratory department with complaints of headache, chest pain, myalgia, fatigue, and anorexia for 7 days.

**Diagnoses:**

Complete blood count showed eosinophilia. The serum was tested showing a positive finding of *A cantonensis* antibody. Cerebrospinal fluid was tested using high-throughput metagenomics analysis and 16 reads for *A cantonensis* were mapped. The patient was diagnosed with *A cantonensis* infection.

**Interventions:**

The patient received a 7-day course of albendazole and 4-day course of prednisone.

**Outcomes:**

When discharged from the hospital, the patient still suffered from fatigue and poor memory. Aminotransferase levels were high due to albendazole’s liver toxicity. In a post-discharge follow-up about 1 month later she had recovered completely both physically and mentally, and peripheral eosinophil count and aminotransferase levels were both normal.

**Lessons:**

Because the direct identification of parasites is difficult, high-throughput metagenomics analysis may provide a reliable alternative tool for the diagnoses of infection with *A cantonensis*. When albendazole is prescribed, caution must be taken with respect to its liver toxicity.

## 1. Introduction

*Angiostrongyliasis cantonensis* was first discovered in the pulmonary arteries and hearts of domestic rats in Guangzhou (Canton), China, by Chen in 1935.^[1]^ *A cantonensis* occasionally causes human angiostrongyliasis manifesting as eosinophilic meningitis or meningoencephalitis.^[2]^ In recent years, with the popularization of high-throughput metagenomics analysis to diagnose diseases of unknown origin or cause, *A cantonensis* was detected in several cases, as *A cantonensis* sequences were found positive in samples from patients.^[3–5]^ Here, we describe a case of human *A cantonensis* with a typical manifestation of eosinophilic meningoencephalitis with the help of high-throughput metagenomics analysis.

**Figure 1.**
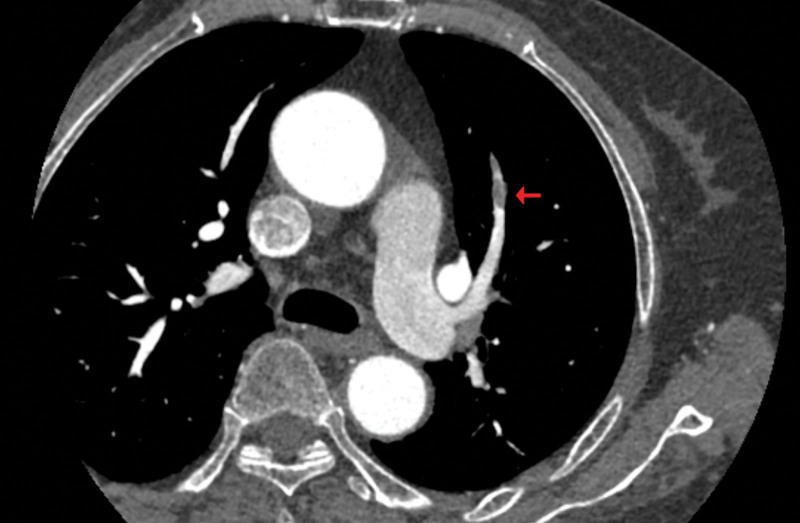
CT pulmonary angiography. Pulmonary thromboembolism in a branch of the left pulmonary artery (red arrow). CT = computed tomography.

A 70-year-old woman patient presented to the respiratory department with compliant headache, chest pain, myalgia, fatigue, and anorexia for 7 days and was admitted to the hospital the next day. In her medical history, a surgery was performed for right nephroureterolithiasis 35 days ago in the same hospital. The vital signs were stable but she had response lag and tenderness when palpated on the head, chest and back. The complete blood count showed an elevated eosinophil count of up to 2.42 × 10^9^/L, accounting for 31% of whole blood white blood cells. The total IgE was 1723 IU/mL (reference range: 1–100 IU/mL) and IgG 20.2 g/L (reference range: 7–16 g/L). The neutrophil count was in the normal range with respect to both the absolute count and the differential percentage. Arterial blood gas analysis revealed hypoxemia with PaO2 58.8 mm Hg and the PaO_2_/FiO_2_ ratio 280 mm Hg. The chest computed tomography (CT) scan showed mild bilateral hydrothorax and the brain CT showed scattered ischemic foci. The D dimer was 4.07 μg/mL (reference range: 0–0.5 μg/mL) and there was pulmonary embolism in the distal pulmonary arterial branches bilaterally by CT angiography (Fig. 1).

After admission to investigate the causes of peripheral eosinophilia, a series of parasite antibodies were tested resulting in a positive finding of the *A cantonensis* antibody in the serum of the patient. None of other common parasite antibody was positive.

Interestingly a review of her past medical history when she received the right nephro-ureterolithiasis surgery about one month ago showed a completely normal eosinophil count and percentage in the complete blood count. Her son provided an additional detail that when discharged from the hospital the patient and her family members got together to have a meal in nearby restaurant and a dish of oncomelania snails was consumed and the patient ate “only one” snail.

*A cantonensis* infection was suspected and then a lumbar puncture was arranged. Cerebrospinal fluid (CSF) was clear in color, but pressure was slightly elevated to 205 mm H_2_O. The pandy test was positive. The total cell count was 327 × 10^6^/L and the differential cytological count showed that eosinophils accounted for 63%. The chlorite concentration was 118.5 mmol/L (reference range: 120–132 mmol/L). The glucose concentration was 2.15 mmol/L. The protein concentration was 919 mg/L (reference range: 120–600 mg/L). No parasite was found with neither naked eyes nor under a microscope.

**Figure 2.**
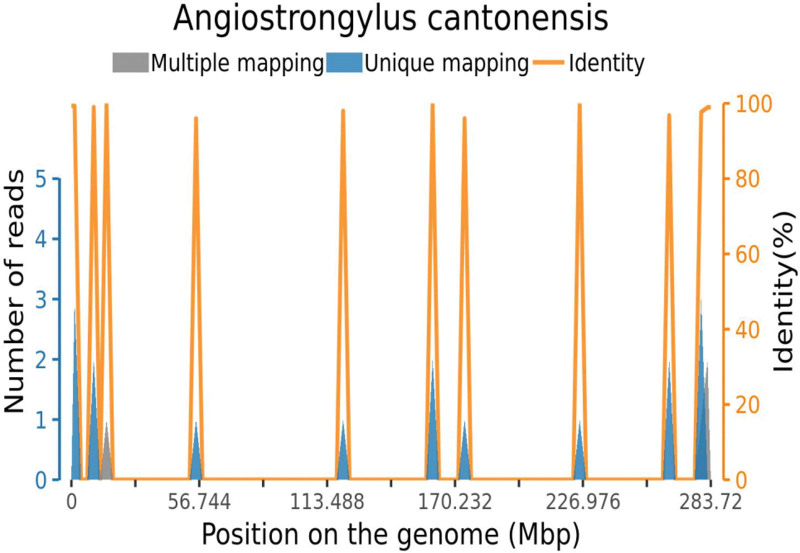
Cover chart of CSF high-throughput metagenomics analysis. With a 0.003% gene coverage and 1.15 read depth for *Angiostrongyliasis cantonensis*, 16 reads of *A cantonensis* were mapped. CSF = cerebrospinal fluid.

The *A cantonensis* antibody in CSF was negative. CSF was tested with high-throughput metagenomics analysis and 16 reads for *A cantonensis* were mapped (Fig. 2). None of the reads of virus, bacteria, fungus, or other parasites were mapped in the CSF.

The final diagnosis was *A cantonensis*. The differential diagnosis included other parasitic infections of the brain that may have manifestation of peripheral and central eosinophilia, but there was on other supportive microbial evidence. Albendazole 200 mg twice a day and prednisone 30 mg once a day orally were prescribed. Albendazole, an antiparasitic agent, and prednisone were chosen to prevent anaphylaxis according to the Chinese guidelines for the diagnosis and treatment of infection with angiostrongyliasis cantonensis.

The patient had a significant improvement in symptoms including neurologic status, myalgia, and appetite the next day. On the sixth day of treatment, the eosinophil count returned to 1.27 × 10^9^/L and the percentage was 17.8% and on the eighth day the eosinophil count was 0.73 × 10^9^/L and the percentage was 13.4%. On day 6 of treatment, alanine transaminase, gamma-glutamyl transferase and aspartate aminotransferase increased to 84 U/L (normal range: 7–40 U/L), 64 U/L (normal range: 13–35 U/L) and 61 U/L (7–45 U/L), respectively. The liver toxicity of albendazole was reported in the literature and usually recovered after discontinuation of albendazole administration.^[6–8]^

**Figure 3.**
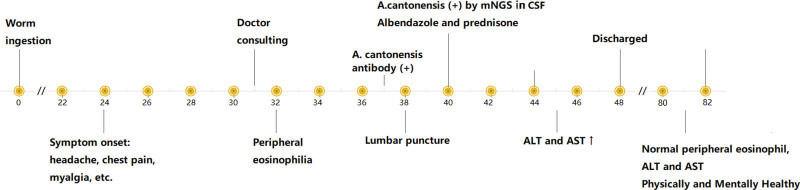
Timeline displaying key events in the case report. ALT = alanine aminotransferase, AST = aspartate aminotransferase, CSF = cerebrospinal fluid.

The patient received a 7 day course of albendazole and a 4 day course of prednisone. When discharged from the hospital, she still suffered from fatigue, unable to stand up, and bad memory. In a post-discharge follow-up about a month later she had recovered completely both physically and mentally, and peripheral eosinophil count and aminotransferase were both normal (Fig. 3).

## 2. Discussion

*A cantonensis* is widely spread from its traditional endemic regions of the Pacific islands and Southeast Asia to the American continent, including the USA, Caribbean islands, and Brazil.^[9]^ Shen Zhen City, the place where this patient lived, about 100 km away from Canton City, is located in a subtropical area that is a favorable habitat for the *A cantonensis* host, such as oncomelania snails. Inhabitants can be exposed to *A cantonensis* by eating intermediate or paratenic hosts, or vegetables contaminated by larvae.^[10]^

This patient had a clear history of exposure as she consumed the oncomelania snail one month ago. This exposure was believed to be causal, as the eosinophil count in the complete blood count one month ago right after surgery was normal. Human susceptibility to *A cantonensis* may vary from person to person, as other family members who ate together kept healthy.

Most human angiostrongyliasis manifests itself as eosinophilic meningitis or meningoencephalitis, the clinical features are quite obvious with respect to peripheral blood and CSF eosinophilia, neurolgical disorders including headache, myalgia, and some nonspecific symptoms such as nausea and fainting.^[2,4,9,10]^ However, a timely diagnosis is not easy to achieve in clinical practice. Delayed diagnosis is quite common, as many patients were not correctly diagnosed until 2 or 3 weeks after hospitalization. The low detection rate is one reason for delayed or even missed diagnosis; In one early literature reported in 1988 in Chinese, the positive detection of *A cantonensis* was less than 2%. A review of several recent case reports of human *A cantonensis* showed that few of these cases reported a positive finding of *A cantonensis* directly.^[4,10–13]^

Angiostrongyliasis is usually considered based on a history of mollusk consumption, clinical characteristics, eosinophilic pleocytosis in the CSF, and the identification of a positive antibody.^[14]^

Infection with *A cantonensis* in humans is only reported sporadically in the literature. It may not be a common disease that physicians can often encounter, which is a barrier for them to make an early and timely diagnosis. Some patients died from the delayed diagnosis of *A cantonensis* infections at a young age who otherwise had a good prognosis if the patient received adequate treatment.^[5,11]^

Peripheral eosinophilia is not specific for *A cantonensis*; however, the eosinophil count test is a component part of the whole blood cell test which is an essential test in the inpatient department, in such circumstances eosinophilia provides an important clue for the diagnosis of a parasite infection that includes *A cantonensis* infection.

This case was diagnosed early and treated quickly. Peripheral eosinophilia provided a vital clue. Eosinophilia can be considered part of drug allergy, as many patients with *A cantonensis* would have received various drugs when the therapeutic efficacy was unsatisfactory,^[10]^ but eosinophilia is almost unexceptionally present in all patients infected with *A cantonensis* at the beginning of the disease.

The *A cantonensis* antibody test is reliable for diagnosis, providing a support for its specificity. However, a false negative result of the *A cantonensis* antibody would delay diagnosis in a timely manner.^[5]^

The high-throughput metagenomics analysis has high sensitivity and specificity for the detection of microorganisms, which has become increasingly popular in recent years. A prospective study reported that the sensitivity of high-throughput metagenomics analysis in the identification of bacteria, fungi, virus and tuberculosis in CSF was 73.3, 80, 76 and 66.7%, respectively, while the specificity of high-throughput metagenomics analysis in the diagnosis of bacteria, fungi and tuberculosis in CSF was 95.9, 79.3 and 96.4%, respectively.^[5,15]^

High-throughput metagenomics analysis is almost a complete representation of the organisms present in a host organism. A positive finding of pathogens from samples from body parts that are otherwise pathogen-free is especially reliable. Sometimes it is a final straw when doctors have a suspicion that a sort of unknown organism might be the etiologic factor but microorganism culture or other routine microbiologic tests fail to detect a pathogen. High-throughput metagenomics analysis can serve as a target-free identification in the diagnosis of rare central nervous system angiostrongyliasis,^[5]^ it can also solidify the diagnosis of *A cantonensis* when traditional examinations are not adequate to make a definitive diagnosis, since high-throughput metagenomics analysis provides direct evidence for the presence of *A cantonensis* nucleic acids.

## Acknowledgments

The authors thank all the clinical and laboratory stuffs contributed in the case.

## Author contributions

**Resources:** Liqun Zhu, Sida Chen, Na Li, Chunxing Ye, Songming Zhuo, Yan Shen.

**Writing – original draft:** Guoqing Qiu.

**Writing – review & editing:** Guoqing Qiu.
